# *Hydrangea serrata* (Thunb.) Ser. Extract Attenuate UVB-Induced Photoaging through MAPK/AP-1 Inactivation in Human Skin Fibroblasts and Hairless Mice

**DOI:** 10.3390/nu11030533

**Published:** 2019-03-01

**Authors:** Hee-Soo Han, Ji-Sun Shin, Da-Bin Myung, Hye Shin Ahn, Sun Hee Lee, Hyoung Ja Kim, Kyung-Tae Lee

**Affiliations:** 1 Department of Pharmaceutical Biochemistry, College of Pharmacy, Kyung Hee University, Seoul 02447, Korea; heesu3620@hanmail.net (H.-S.H.); jsunvet@naver.com (J.-S.S.); dabin_happy@naver.com (D.-B.M.); 2 Department of Life and Nanopharmaceutical Science, College of Pharmacy, Kyung Hee University, Seoul 02447, Korea; 3 Department of New Material Development, COSMAXBIO, Seongnam 13486, Korea; ztztzt08@naver.com (H.S.A.); bt-shlee@cosmax.com (S.H.L.); 4 Molecular Recognition Research Center, Materials and Life Science Research Division, Korea Institute of Science and Technology, Seoul 02792, Korea; khj@kist.re.kr

**Keywords:** *Hydrangea serrata* (Thunb.) Ser., ultraviolet B, photoaging, MMPs, collagen, MAPK, AP-1

## Abstract

Skin photoaging is mainly caused by exposure to ultraviolet (UV) light, which increases expressions of matrix metalloproteinases (MMPs) and destroys collagen fibers, consequently inducing wrinkle formation. Nutritional factors have received scientific attention for use as agents for normal skin functions. The aim of this study was to investigate the effect of hot water extracts from the leaves of *Hydrangea serrata* (Thunb.) Ser. (WHS) against ultraviolet B (UVB)-induced skin photoaging and to elucidate the underlying molecular mechanisms in human foreskin fibroblasts (Hs68) and HR-1 hairless mice. WHS recovered UVB-reduced cell viability and ameliorated oxidative stress by inhibiting intracellular reactive oxygen species (ROS) generation in Hs68 cells. WHS rescued UVB-induced collagen degradation by suppressing MMP expression, and reduced the mRNA levels of inflammatory cytokines. These anti-photoaging activities of WHS were associated with inhibition of the activator protein 1 (AP-1), signal transduction and activation of transcription 1 (STAT1), and mitogen-activated protein kinase (MAPK) signaling pathways. Oral administration of WHS effectively alleviated dorsal skin from wrinkle formation, epidermal thickening, collagen degradation, and skin dehydration in HR-1 hairless mice exposed to UVB. Notably, WHS suppressed UVB activation of the AP-1 and MAPK signaling pathways in dorsal mouse skin tissues. Taken together, our data indicate that WHS prevents UVB-induced skin damage due to collagen degradation and MMP activation via inactivation of MAPK/AP-1 signaling pathway.

## 1. Introduction

The skin, as the most vulnerable organ of the human body, functions as the first line of defense protecting the body against toxic chemicals, infections, and ultraviolet (UV) radiation [1]. The process of skin aging is classified either as intrinsic (chronological) or extrinsic (photoaging). Intrinsic skin aging is a natural process and is typically influenced by genetic factors. In contrast, extrinsic aging is mainly caused by repeated exposure to solar ultraviolet light, especially UVB, and is characterized by a thickened epidermis, mottled discoloration, deep wrinkles, loss of elasticity, and slowing of skin cell growth associated with slower wound healing [2]. Excessive UVB irradiation penetrates the skin and generates intracellular reactive oxygen species (ROS), which consequently results in cellular oxidative stress and prolonged skin inflammation [3] through activation of mitogen-activated protein kinase (MAPK), activator protein 1 (AP-1), nuclear factor kappa B (NF-κB)/p65, and signal transduction and activation of transcription (STAT) pathways [4]. Activation of these pathways results in overexpression of inflammatory cytokines like tumor necrosis factor (TNF)-α, interleukin (IL)-1β, and IL-6 [5]. Moreover, UVB-induced ROS upregulates the expression of matrix metalloproteinases (MMPs), contributing to extracellular matrix (ECM) degradation, including the destruction of collagen fibers, and consequently resulting in wrinkle formation [6,7]. Increased levels of MMPs and degradation of ECM components such as collagen, hyaluronic acid (HA), and elastin, are thought to be prominent characteristics of photo-damaged skin [8].

Several studies have indicated that dietary ingredient supplementation may improve or delay skin aging [9,10]. Various chemicals or medicines proposed to alleviate skin aging have disadvantages, including high prices, chemical instability, and side effects [11]. Therefore, there has been growing interest in the development of safe and effective materials from botanical sources to prevent or treat damaged skin. *Hydrangea* (Hydrangeaceae) leaves have been consumed as a tea and as medicine in far-east Asian countries like Korea, China, and Japan [12]. Several studies have demonstrated that extracts or isolated compounds of hydrangea leaves possess anti-inflammatory [13], anti-diabetic [14], renal protective [15], and hepatoprotective activities [16] through an ameliorating effect on oxidative stress. *Hydrangea serrata* (Thunb.) Ser. is native to the Korean mountains known as “San-soogook, Mountain Hydrangea, or Tea of Heaven”. Its leaves are used to make herbal teas. Our recent phytochemical study showed that a variety of compounds isolated from *H. serrata* possess anti-photoaging activity in UVB-exposed human fibroblasts. Among these compounds, hydrangenol has potential protective effects on cell viability, production of procollagen type I, MMP-1, and pro-inflammatory cytokines [17]. Based on our previous report, we concluded that *H. serrata* may have the potential for application as a beneficial natural anti-photoaging agent. However, due to the lack of evidence on the anti-photoaging activity of *H. serrata* extract per se, we investigated the anti-photoaging effect of hot water extracts from the leaves of *H. serrata* (WHS) in UVB-irradiated Hs68 human fibroblasts and HR-1 hairless mice.

## 2. Materials and Methods

### 2.1. Preparation and Standardization of WHS

*Hydrangea serrata* (Thunb.) Ser. were cultivated in Yeongju, Korea and were identified by Dr. Hyung-Jun Kim from Forest Medicinal Resources Research Center in National Institute of Forest Science (NIFoS, Seoul, Korea). A specimen voucher has been deposited in COSMAXBIO (COSMAXBIO, Seongnam, Korea). The dried leaves of *H. serrata* (1000 g) were extracted with distilled water at 98 °C for 5 h followed by filtration and then spray-dried to give a dried extract residue WHS (261 g, 26.1%). The HPLC system consisted of a Waters model 2695 HPLC pump with photodiode array detector (Waters model 2998) set to 228 nm (Waters, Milford, MA, USA). Separations were achieved at 30 °C using a Luna C18 (4.6 mm, 250 mm, I.D., 5 μm, Phenomenex) column. The gradient mobile phases consisted of solvent A (100% acetonitrile) and solvent B (water) with a gradient elution as follows: 0 to 15 min, 20% to 25%; 15 to 30 min, 25% to 50%; 30 to 40 min, 50% to 100%; 40 to 50 min, 100% to 20% as percent of solvent A at a flow rate of 1.0 mL/min.

### 2.2. Cell Culture

Human fibroblasts (Hs68) were obtained from the American Type Culture Collection (ATCC, Manassas, VA, USA) and cultured in Dulbecco’s modified eagle’s medium (DMEM) containing 10% fetal bovine serum (FBS), penicillin-streptomycin sulfate (100 U/mL and 100 μg/mL) at 37 °C with 5% CO_2_. Hs68 cells were plated at 1 × 10^5^/mL in culture area. After 24 h incubation, the cells were washed once with PBS and then exposed to UVB irradiation (15 mJ/cm^2^) using UVP Crosslinker (Analytik Jena AG, Jena, Germany). After UVB irradiation, cells were incubated in fresh culture media in the presence or absence of WHS (6.25, 12.5, or 25 μg/mL).

### 2.3. Determination of Cell Viability

After UVB irradiation, Hs68 cells were treated with WHS for 24 h. Cell viability was estimated using 3-(4,5-Dimethylthiazol-2-yl)-2,5-diphenyl tetrazolium bromide (MTT) assay. 20 μL MTT solution (5 mg/mL) was added to each well, and the cells were further incubated for an additional 4 h. The supernatant was removed and the formazan was resolved with 1 mL/well of DMSO. The optical density was measured by microplate reader (Molecular Devices Inc., San Jose, CA, USA) at 540 nm.

### 2.4. Fluorescence Assay of Intracellular ROS

After UVB irradiation, Hs68 cells were treated with WHS (6.25, 12.5, or 25 μg/mL) or *N*-acetyl-l-cysteine (NAC, 10 mM) for 24 h. For measuring intracellular ROS generations, the cells were stained with 20 μM H_2_DCFDA for 30 min. The stained cells were resuspended in PBS and fluorescence intensities were detected by flow cytometry (Beckman Coulter Inc., Brea, CA, USA).

### 2.5. Elastase Activity Assay

Elastase activity was measured using *N*-succinyl-tri-alanyl-*p*-nitroanilide (STANA, elastase substrate) as previously reported, with some modifications [18]. Cells were collected and dissolved in 0.1% Triton X-110 in 0.2 M Tris-HCl buffer solution (pH 8.0). The cell resuspension was frozen and thawed for three times. Cell debris was removed by microcentrifugation (3000 rpm, 20 min, 4 °C). 100 μg of each cellular protein was loaded in 96-well plates and the volume of each well was adjusted with 0.2 M Tris-HCl buffer by 98 μL. 2 μL of 50 mM *N*-succinyl-tri-alanyl-*p*-nitroanilide (elastase substrate) solution was dispensed into each well and then incubated for 90 min at 37 °C. The elastase activity was determined by measuring the absorbance of each well at 405 nm using a microplate reader (Molecular Devices Inc., San Jose, CA, USA).

### 2.6. Measurement of Pro-Collagen Type I, MMPs, and HA Production

The production of pro-collagen type I (cat#. MK101) and MMP-1/-3 (cat#. ab100603/ab100607) in cell culture media and the levels of hyaluronic acid (HA) (cat#. DY3614-05) in skin tissues were quantified by ELISA kits according to the manufacturer’s instructions (TaKaRa Bio Inc., Shiga, Japan; Abcam, Cambridge, UK; and R&D Systems, Minneapolis, MN, USA, respectively).

### 2.7. RNA Extraction and Quantitative Real-Time RT-PCR (qRT-PCR)

Total cellular RNA was extracted by using Easy Blue^®^ kits (Intron Biotechnology, Seoul, Korea). RNA (1 μg) was reverse-transcribed (RT) using 0.5 mg/mL random oligonucleotide primers (Promega, Madison, WI, USA) and TOPscript^TM^ RT DryMIX (Enzynomics, Daejeon, Korea). PCR amplification was performed using the incorporation of SYBR green using SYBR Primix Ex Taq (TaKaRa Bio Inc., Shiga, Japan). The PCR primers used in this study are described in Table S1. Steady-state mRNA levels were determined by real time qPCR using the TaKaRa thermal cycler device. Mean Ct values of genes were calculated from triplicate measurements and normalized versus the mean Ct of GAPDH. The PCR primers used in this study are described in Table S1.

### 2.8. Western Blot Analysis

Total cellular or nuclear protein extracts from UVB-treated or UVB plus WHS-treated cells were prepared as described previously [19]. Proteins (25 to 50 μg) were resolved by SDS-PAGE on 8% to 12% polyacrylamide gel and electrotransferred to polyvinylidene fluoride (PVDF) membrane. The immunoblot was incubated in blocking solution (5% skim milk) for 1 h at room temperature and then incubated overnight with a 1:1000 dilution of primary antibody at 4 °C. The blots were washed three times with tris-buffered saline with 0.1% tween 20 (TBST), incubated with a 1:2000 dilution of horseradish peroxidase-conjugated secondary antibody for 2 h at room temperature, washed again three times with TBST, and finally developed using an enhanced chemiluminescence (ECL) substrate (Santa Cruz Biotechnology, Santa cruz, CA, USA). The protein bands were visualized by LAS-4000 luminescent image analyzer (FUJIFILM, Tokyo, Japan). The antibodies used in this study are listed in Table S2. The optical density of each representative blot was presented on the basis of internal control proteins (Histone H3 or β-actin).

### 2.9. Animals

Five-week-old male HR-1 hairless mice were purchased from SLC Inc. (SLC Inc., Shizuoka, Japan). The animals were housed under consistent conditions (temperature: 22 ± 1 °C, humidity: 40% to 60%, light/dark cycle: 12 h). All experiments were conducted under university guidelines of ethical committee for Animal Care and Use of the Kyung Hee University according to an animal protocol (KHUASP (SE) -18-107). After the acclimation period (1 week), mice were randomly divided into five groups (*n* = 8): Vehicle-treated control (without UVB irradiation), UVB only-treated, UVB + WHS (25 mg/kg/day, p.o.), UVB + WHS (50 mg/kg/day, p.o.), and UVB + WHS (100 mg/kg/day, p.o.).

### 2.10. UVB-Irradiated Skin Aging Model

UVB irradiation was applied to the backs of mice, three times a week during 10 weeks using UVP Croslinker (Analytik Jena AG, Jena, Germany). The energy of UVB was progressively increased from 60 mJ/cm^2^ at first 4 weeks, to 240 mJ/cm^2^ at final irradiation with regular intervals of 60 mJ/cm^2^ for every 2 weeks.

### 2.11. Evaluation of Skin Wrinkle Formation

The skin wrinkle formation on the dorsal skin of mice was observed at 10 weeks shortly after a sacrifice. The skin replicas were cast on the dorsal skin surface of mice using SILFLO (Flexico developments LTD., Tokyo, Japan) and then analyzed to measure skin wrinkles (Visioline^®^ VL 650). The parameters for the assessment of skin wrinkles were total wrinkle area, mean length, mean depth, and max wrinkle depth.

### 2.12. Histological Analysis

The dorsal skin specimens were obtained, fixed in 4% paraformaldehyde overnight, dehydrated in ethanol and then embedded in paraffin. The sliced sections were stained with hematoxylin and eosin (H&E) for skin layers and Masson’s trichrome for collagen fiber analysis at the Seoul Medical Science Institute (SCL. Co. Ltd., Seoul, Korea).

### 2.13. Physiological Analysis of the Skin Surface

On the day of sacrifice, the dorsal skin thickness of the mice was measured using a caliper. Skin hydration and skin transepidermal water loss (TEWL) on the back of the mice were measured using a GPSkin Barrier^®^ (GPOWER Inc., Seoul, Korea) [20]. The instrument’s probe tip is designed as a closed chamber with a water loss sensor on the top and two skin hydration sensors on the edge of the chamber. The values of skin hydration and TEWL were automatically calculated and expressed in arbitrary units (AU) and g/m^2^/h, respectively.

### 2.14. Statistical Analysis

Values are presented as the mean ± SD of triplicate experiments (in vitro). In the animal study, data were expressed as the mean ± SD (*n* = 8 for skin aging indicators, *n* = 5 for qRT-PCR, and *n* = 3 for Western blotting). Comparison between groups was made by using analysis of variance and Dunnett’s post hoc test. *p*-values of 0.05 or less were considered statistically significant.

## 3. Results

### 3.1. Identification of Active Compounds from WHS

Previously, using Hs68 fibroblasts, we showed that hydrangenol is a valuable skin protective compound against UVB-induced skin damage [17]. To understand the anti-photoaging activity of WHS, we first standardized WHS with hydrangenol and then performed a chromatographic analysis using an HPLC/UV system with an analytical C_18_ column. As shown in Figure S1, a hydrangenol peak was identified in WHS at 33.07 min compared to the hydrangenol standard. The proportion of hydrangenol in WHS was recorded as 0.63 ± 0.05%.

### 3.2. WHS Attenuates UVB-Reduced Cell Proliferation, UVB-Induced ROS Generation, Elastase Activity, and Degradation of Collagen in Hs68 Fibroblasts

**Figure 1:**
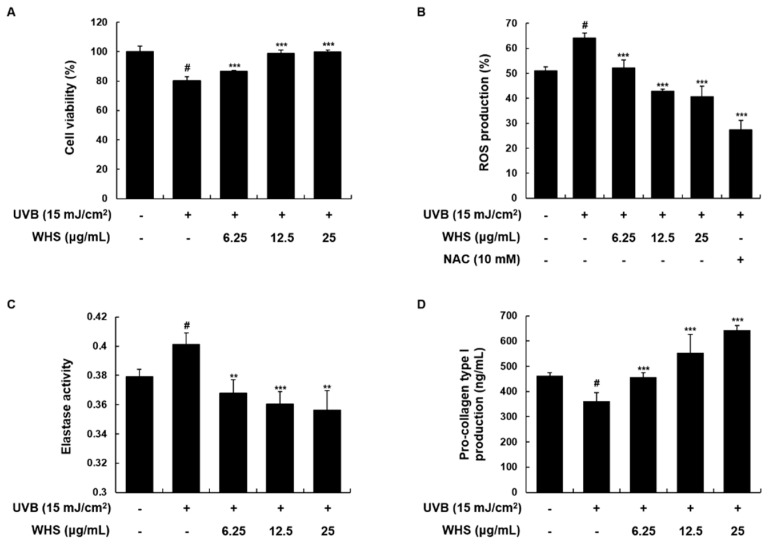
Effects of *Hydrangea serrata* (Thunb.) Ser. (WHS) on cell proliferation, reactive oxygen species (ROS) generation, elastase activity, and procollagen type I production in UVB-induced Hs68 fibroblasts. Cells were exposed to UVB irradiation (15 mJ/cm^2^) and then treated with WHS (6.25, 12.5, or 25 μg/mL) for 24 h. (**A**) Cell viability was evaluated with an MTT assay. (**B**) After incubation, cells were stained with H_2_DCFDA (20 μM) for 30 min. ROS levels were evaluated by flow cytometry. *N*-acetyl-l-cystein (NAC) (10 mM), a common ROS inhibitor, was used as a positive control. (**C**) Cellular protein was prepared to examine elastase activity, determined using the elastase substrate STANA in WHS-treated cells. (**D**) The cell culture media were collected to determine the levels of procollagen type I. Values are expressed as means ± SD of three independent experiments. ^#^ *p* < 0.05 vs. the control group; ** *p* < 0.01, and *** *p* < 0.001 as compared to the UVB-irradiated group.

We initially conducted an MTT assay to investigate the cytoprotective effect of WHS in UVB-induced Hs68 fibroblasts. As shown in Figure 1A, UVB exposure (15 mJ/cm^2^) reduced cell viability by 80.18 ± 2.57% compared to control cells; however, WHS diminished the decrease of cell proliferation induced by UVB (86.75 ± 0.46% at 6.25 μg/mL, 98.65 ± 2.66% at 12.5 μg/mL, and 99.69 ± 1.51% at 25 μg/mL). As shown in Figure 1B, UVB induced an increase in ROS generation, which was significantly suppressed by WHS treatment. We also evaluated the effect of WHS treatment on elastase activity and procollagen type I production. As shown in Figure 1C, WHS treatment significantly reduced the UVB-induced elastase activity, suggesting that the WHS inhibited against elastin degradation. Cells exposed to UVB exhibited markedly reduced procollagen type I production to 78.27 ± 7.59%. Production of procollagen type I was significantly improved with WHS treatment (99.07 ± 3.65% at 6.25 μg/mL, 119.8 ± 15.83% at 12.5 μg/mL, and 139.26 ± 3.95% at 25 μg/mL; Figure 1D).

### 3.3. WHS Attenuates UVB-Induced MMP-1/-3 Production and mRNA Expression, as Well as Skin Inflammation, in Hs68 Fibroblasts

**Figure 2:**
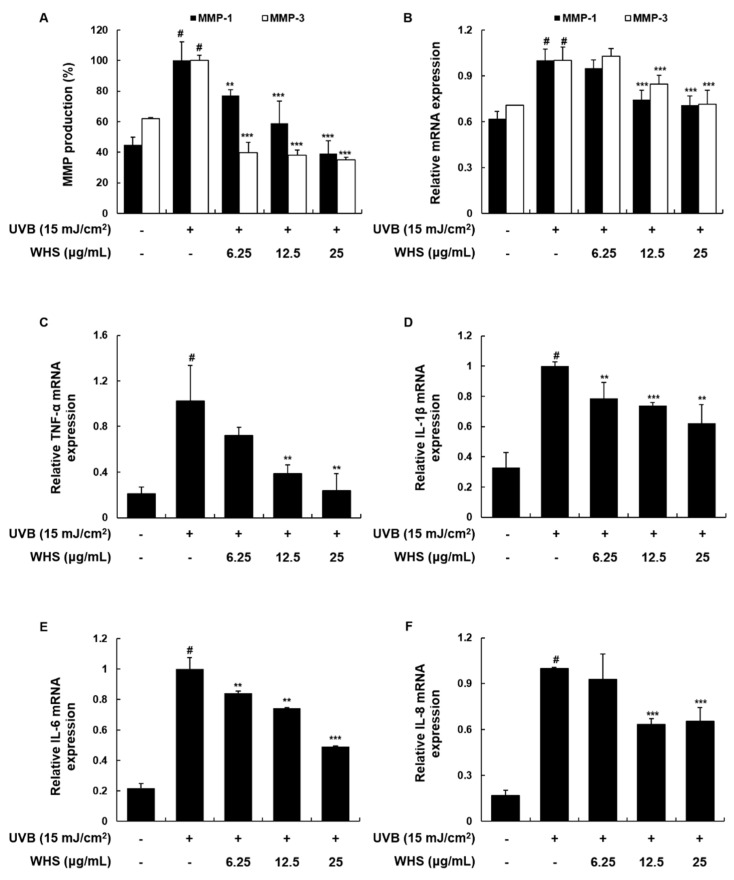
Effects of WHS on MMP-1/-3 protein and mRNA expression levels, and on mRNA expression of pro-inflammatory cytokines in UVB-exposed Hs68 fibroblasts. Cells were exposed to UVB irradiation (15 mJ/cm^2^) prior to WHS treatment (6.25, 12.5, or 25 μg/mL). (**A**) Following a 24 h incubation, the cell culture media were collected to determine MMP levels, using ELISA kits. (**B**–**F**) Following a 3 h incubation, total cellular RNA was extracted from WHS-treated cells. The mRNA levels of MMPs, TNF-α, IL-1β, IL-6, and IL-8 were quantified by qRT-PCR and adjusted to GAPDH expression. Values are expressed as means ± SD of three independent experiments. ^#^ *p* < 0.05 vs. the control group; ** *p* < 0.01, and *** *p* < 0.001 as compared to the UVB-irradiated group.

Since MMPs are the major endopeptidases inducing collagen degradation, overexpression of MMPs is a major characteristic of photo-damaged skin [21,22]. We found that MMP-1/-3 production and mRNA expression were both increased by UVB irradiation, but WHS significantly inhibited these upregulations (Figure 2A,B). Irradiation with UVB also has the potential to induce pro-inflammatory cytokines that contribute to skin inflammation [23]. We then investigated the effect of WHS on the mRNA levels of pro-inflammatory cytokines by qRT-PCR. As shown in Figure 2C–F, WHS (25 μg/mL) significantly downregulated UVB-induced mRNA expression levels of TNF-α, IL-1β, IL-6, and IL-8 by 23.67 ± 15.12%, 62.29 ± 12.14%, 49.26 ± 0.48%, and 65.45 ± 8.95%, respectively, compared to that in cells exposed only to UVB.

### 3.4. WHS Inhibits the Activation of the AP-1, STAT1, and MAPK Signaling Pathways in UVB-Exposed Hs68 Fibroblasts

**Figure 3:**
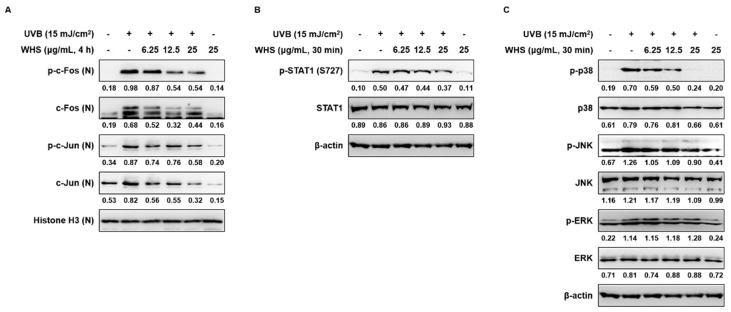
Effects of WHS on the activator protein 1 (AP-1), signal transduction and activation of transcription 1 (STAT1), and mitogen-activated protein kinase (MAPK) signaling pathways in UVB-exposed Hs68 fibroblasts. Cells were irradiated with UVB (15 mJ/cm^2^) and then treated with WHS (6.25, 12.5, or 25 μg/mL) for 4 h (AP-1) or 30 min (STAT1 and MAPK). (**A**) Nuclear or (**B**,**C**) total cellular protein was prepared and resolved by SDS-PAGE, transferred to PVDF membranes, and detected with specific p-c-Fos (Ser32), p-c-Jun (Ser63), p-STAT1 (S727), p-p38, p-JNK, and p-ERK antibodies. Histone H3 and β-actin were used as internal controls for nuclear and whole-cell lysates, respectively. Presented data are the representative blots of three independent experiments.

Irradiation with UVB has been reported to activate AP-1 and its upstream regulators, MAPKs, thus accelerating MMP transcription and skin inflammation [24]. Our results showed that UVB induced the phosphorylation and expression of the AP-1 subunits c-Fos and c-Jun, as well as the phosphorylation of signal transduction and activation of transcription 1 (STAT1) at Ser727, but WHS effectively inhibited these effects (Figure 3A,B). Moreover, UVB stimulated overall MAPK signaling, and WHS suppressed the phosphorylation of p38 and c-Jun N-terminal kinases (JNK), but not extracellular signal-regulated kinase (ERK) (Figure 3C).

### 3.5. WHS Ameliorates UVB-Induced Wrinkle Formation on the Dorsal Skin of HR-1 Hairless Mice

**Figure 4:**
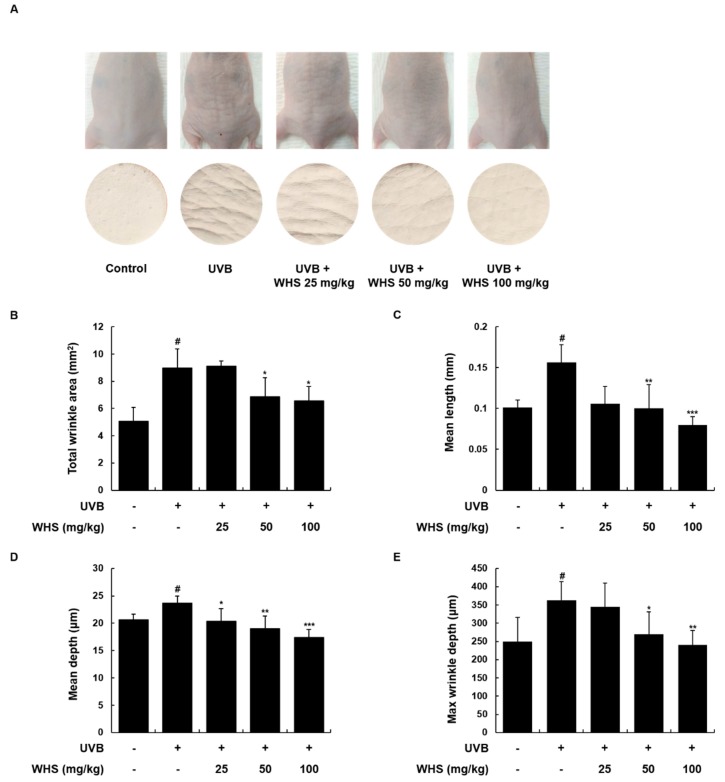
Effects of WHS on skin wrinkle formation in UVB-irradiated hairless mice. HR-1 hairless mice were orally administrated WHS (25, 50, or 100 mg/kg) daily and exposed to UVB irradiation three times a week for 10 weeks. The skin replica samples of the dorsal areas were taken shortly after sacrifice. (**A**) Representative examples of external appearance of dorsal skin surface. The parameters for wrinkle formation, including (**B**) total wrinkle area, (**C**) mean length, (**D**) mean depth, and (**E**) maximum wrinkle depth, were obtained from the skin replica analysis. Values are expressed as means ± SD (*n* = 8). ^#^ *p* < 0.05 vs. the vehicle-treated control group; * *p* < 0.05, ** *p* < 0.01, and *** *p* < 0.001 as compared to the UVB only-treated group.

To verify the anti-photoaging effect of WHS in vivo, we used UVB-irradiated HR-1 hairless mice. We examined the alleviative effect of WHS on UVB-induced wrinkle formation by observing the photographs and skin replicas of the mice (Figure 4). The repetitive and gradual increase of UVB exposure induced significant wrinkle formation in the dorsal skin. However, treatment with WHS (25, 50, or 100 mg/kg; p.o.) potently reduced the wrinkle formation induced by UVB irradiation. Skin wrinkle severity, including total wrinkle area, mean length and depth of wrinkles, and maximum wrinkle depth, were quantified by analysis of the skin replicas. These wrinkle grade biomarkers significantly increased in the UVB only-treated group, whereas they dose-dependently decreased in the UVB + WHS-treated groups (Figure 4B–E).

### 3.6. WHS Attenuates UVB-Induced Skin Thickening and Restores Collagen Synthesis, HA Production, and Loss of Water Content on the Dorsal Skin of HR-1 Hairless Mice

**Figure 5:**
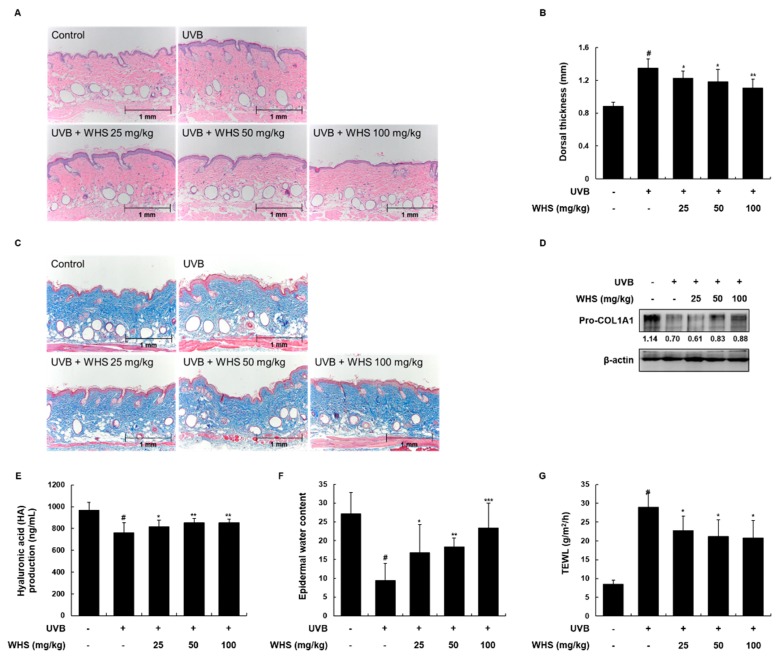
Effects of WHS on skin thickness and skin moisturizing factors in UVB-irradiated hairless mice. HR-1 hairless mice were orally administrated WHS (25, 50, or 100 mg/kg) daily and exposed to UVB irradiation three times a week for 10 weeks. (**A**) Skin tissues were stained with hematoxylin and eosin (H&E) to evaluate epidermal thickness and (**B**) the dorsal thickness of the skin of hairless mice was measured with a caliper before sacrifice. (**C**) Collagen fibers were identified with Masson’s trichrome staining. Skin tissues were homogenized for protein extraction. (**D**) Representative blots of pro-COL1A1 protein expression (*n* = 3). (**E**) Hyaluronic acid (HA) production was measured with the lysates using ELISA kits. (**F**) Skin hydration and (**G**) transepidermal water loss (TEWL) were measured in the back of the mice. Values are expressed as means ± SD (*n* = 8). ^#^ *p* < 0.05 vs the vehicle-treated control group; * *p* < 0.05, ** *p* < 0.01, and *** *p* < 0.001 as compared to the UVB only-treated group.

We further examined the effect of WHS on the skin thickness of mice as another biomarker of skin photoaging. Changes in epidermal thickness were evaluated by H&E staining. The dorsal thickness from the skin of mice in the UVB only-treated group increased 1.53-fold compared to that of the vehicle-treated control group, and was effectively reduced by 0.91, 0.88, and 0.82-fold following oral administration of WHS (25, 50, or 100 mg/kg, respectively) compared to the UVB only-treated group (Figure 5A,B). We evaluated collagen fiber organization, HA production, skin hydration, and transepidermal water loss (TEWL) as skin moisturizing factors. As shown in Figure 5C, WHS treatment rescued the UVB-induced spatial density reduction of the collagen fiber network. The UVB-induced reduction of pro-COL1A1 expression and HA production in dorsal skin tissues was significantly inhibited by WHS administration (Figure 5D,E). Moreover, administration of WHS significantly restored the epidermal water content and TEWL (Figure 5F,G).

### 3.7. WHS Attenuates the Increased MMP-1/-3 Protein Levels and IL-1β and IL-6 mRNA Expression Levels by Inhibiting the AP-1 and MAPK Signaling Pathways in UVB-Exposed HR-1 Hairless Mice

**Figure 6:**
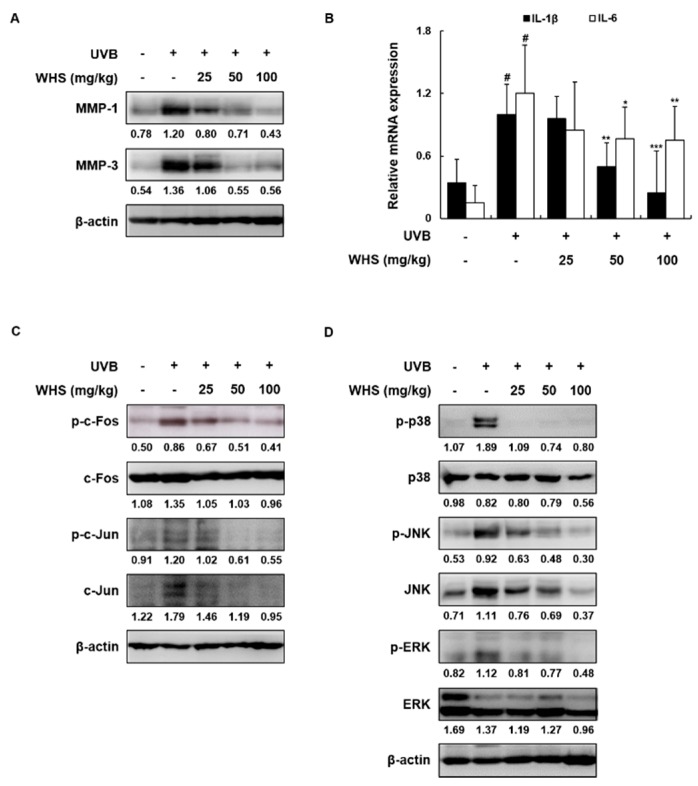
Effects of WHS on protein expression, pro-inflammatory cytokine mRNA levels, and the AP-1 and MAPK signaling pathways in UVB-irradiated hairless mice. HR-1 hairless mice were orally administrated WHS (25, 50, or 100 mg/kg) daily and exposed to UVB irradiation three times a week for 10 weeks. Skin tissues were homogenized for protein or RNA extraction. (**A**) Representative blots of MMP-1/-3 protein expression (*n* = 3). (**B**) The mRNA levels of IL-1β and IL-6 were quantified by qRT-PCR and adjusted to β-actin expression (*n* = 5). Representative blots of (**C**) AP-1 and (**D**) MAPK signaling pathway (*n* = 3). The protein lysates were resolved by SDS-PAGE, transferred to PVDF membranes, and detected with specific MMP-1/-3, p-c-Fos, p-c-Jun, p-p38, p-JNK, and p-ERK antibodies. β-actin was used as the internal control for total protein lysates. Values are expressed as means ± SD. ^#^ *p* < 0.05 vs. the vehicle-treated control group; * *p* < 0.05, ** *p* < 0.01, and *** *p* < 0.001 as compared to the UVB only-treated group.

In accordance with the in vitro data, we examined the levels of MMP-1/-3 and pro-inflammatory cytokines. The protein levels of MMP-1/-3 and the mRNA expression levels of IL-1β and IL-6 were elevated in the UVB only-treated group compared to the vehicle-treated control group, but treatment with WHS effectively attenuated these increases (Figure 6A,B). As shown in Figure 6C, exposure to UVB enhanced c-Fos and c-Jun phosphorylation, which was significantly suppressed by WHS administration. Interestingly, WHS inhibited c-Fos phosphorylation with unchanged total c-Fos expression, but inhibition of c-Jun phosphorylation by WHS resulted from the downregulation of total c-Jun expression. We then examined whether WHS affects UVB-induced activation of MAPKs (p38, JNK, and ERK), the upstream regulators of AP-1, and found that the UVB-induced phosphorylation of p38, ERK, and JNK was markedly suppressed by WHS. Interestingly, decreased JNK phosphorylation resulted from the down-regulation of JNK protein expression (Figure 6D).

## 4. Discussion

With the increase in the average human lifespan, the functional and physical skin changes that accompany the aging process have received growing attention. Studies have shown that certain dietary factors can improve the signs of skin aging [9,25]. Accordingly, the attention of the cosmetic industry has recently focused on numerous crude herbal extracts to prevent skin aging [26,27]. In this report, and for the first time, we aimed to determine the anti-photoaging activity of WHS and elucidate the associated molecular mechanisms.

We used different regimes of UVB exposure and WHS treatment for in vitro and in vivo experiments (in vitro; a single UVB exposure and post-incubated with WHS, in vivo; periodic UVB exposure and daily administration of WHS) by reference to previous reports [7,11,28,29]. Because the cultured fibroblasts were grown in a single layer and directly exposed to UVB, the photoaging reactions were well-induced even in UVB single exposure. In contrast, the skin of mice is composed of epidermis and dermis including various kinds of skin cells. The photo-damaged skin could only be established by repeated exposure to UVB in mice, hence WHS were administered daily to validate its efficacy on the skin aging.

Irradiation with UVB induces cytotoxicity by increasing ROS production, which triggers the skin damage process [30]. In this study, we found that WHS not only rescued the UVB-induced cytotoxicity, but also substantially inhibited intracellular ROS generation in human fibroblasts. Together with elastin and hyaluronic acid, procollagen type I, a major protein component of the ECM, is responsible for skin elasticity and moisture. Normal collagen turnover is regulated by MMPs [8]. Among the different MMPs, MMP-1, a collagenase, and MMP-3, an activator of proMMP-1, are two main contributors to the collagen degradation process [31]. In our study, UVB exposure altered the expression of MMP-1/-3 and procollagen type I in human fibroblasts, while WHS notably reversed these patterns.

The molecular mechanisms of skin aging are complex signaling cascades mostly initiated by UVB-induced ROS, which subsequently activate various intracellular transcription factors, including AP-1, NF-κB, and STATs [7]. AP-1 is a major regulatory protein consisting of two subunits, c-Fos and c-Jun, and is strongly implicated in mediating the photoaging response [32]. It was also reported that there is an AP-1 binding sites in the regulatory sites of MMP genes. Several growth factors and cytokines stimulate the expression of AP-1 transcription factors (c-fos and c-jun), bind to the AP-1 binding site of MMP genes and activate their gene expression [8,33]; STAT1 is an additional transcription factor controlling UVB-induced stress signals and skin inflammation [34]. These transcription factors not only regulate UVB-induced MMP expression, but also mediate skin inflammation responses by increasing the production of pro-inflammatory cytokines [31,33]. It has also been described that inhibition of MAPKs using specific inhibitors (SB202190 or SB203580, p38 inhibitor; U0126 or PD98059, ERK inhibitor; SP600125, JNK inhibitor) down-regulated the UVB-induced AP-1 activation and MMP-1 expression [35,36,37,38]. MAPKs, which include three subgroups (p38, JNK, and ERK), play a central role in mediating the transduction of a series of distinct intracellular events that control various downstream transcription factors, including AP-1 and STATs [39]. Previous reports have described that activation of the AP-1 signaling pathway is regulated by MAPK [40], moreover, p38 has been shown to be required for the stress-induced phosphorylation of STAT1 at Ser727 [41]. Here, we revealed that WHS significantly decreased the UVB-stimulated phosphorylation and nuclear expression of c-Fos and c-Jun, as well as the phosphorylation of STAT1 (Ser727). In addition, treatment with WHS suppressed the UVB-induced phosphorylation of p38 and JNK, but not ERK. It is well known that p38 and JNK respond strongly to inflammation or stress signals [39]. In contrast, ERK signaling is involved in transmitting signals activated by growth factors and regulates cell growth, survival, and differentiation [42]. Thus, our results suggest that WHS mainly contributes to the inhibition of inflammatory signals in cells exposed to UVB-triggered cellular stress.

To investigate the effect of WHS on photo-damaged skin in vivo, we repeatedly irradiated HR-1 hairless mice with UVB. We initially investigated the acute toxicity of WHS in mice prior to evaluating its anti-photoaging properties. In a toxicity test, at doses between 800 mg/kg and 2500 mg/kg (p.o.), WHS did not elicit mortality, gross behavioral changes, or toxic symptoms in organs during a 14 day observational period. This suggests that WHS is not toxic in vivo at the administered doses, and that the approximate lethal dose is higher than 2500 mg/kg. In the animal studies, we used up to 1/25th of this dose (25 to 100 mg/kg) based on the toxicity reported in previous in vivo experiments [14,43]. Ultraviolet rays penetrate the skin in a wavelength-dependent manner: UVA (long wavelength; 320 to 400 nm) deeply penetrates the dermis, while UVB (short wavelength; 290 to 320 nm) is mostly absorbed by the epidermis, damaging the superficial epidermal layers [44]. Consistent with this, our results showed that UVB exposure caused thickening of the epidermis in mice, while the dermis remained unchanged. Irradiation with UVB accelerated wrinkle formation and skin dehydration, whereas oral administration of WHS potently diminished epidermal thickness, wrinkling, and TEWL in the dorsal skin of mice. Treatment with WHS also rescued UVB-induced collagen degradation and reduction of skin moisturizing factors, including HA production and epidermal water content of the dorsal skin of mice. According to our previous research, WHS contains various types of chemical ingredients, with hydrangenol having been identified as a main active component of WHS, possessing the most effective anti-photoaging properties [17]. Hydrangenol and its derivatives have also been shown to effectively inhibit skin conditions, such as allergic [45] or passive cutaneous anaphylaxis reaction [46]. Based on these previous results, we speculate that hydrangenol is the most active component of WHS for improving damaged skin.

UVB can induce DNA damage by producing DNA photoproducts such as cyclobutane pyrimidine dimers (CPD) and pyrimidine (6-4) pyrimidone photoproducts [47]. These mutagenic and cytotoxic DNA lesions can cause severe structural distortions in DNA molecules and limit vital cellular processes including DNA replication and transcription, which subsequently lead to mutagenesis, carcinogenesis, and cell death [48]. Some antioxidant compounds have been reported to protect against UVB-induced DNA damage by inhibiting the formation of these photoproducts [49,50,51]. To elucidate the photoprotective effect of WHS against UVB damage, it needs to be further studied whether WHS influences enhanced DNA repair or inhibits DNA damage.

## 5. Conclusions

In summary, our in vitro and in vivo experiments showed that WHS effectively prevents skin photoaging by enhancing collagen deposition and inhibiting MMPs and inflammatory cytokines via the MAPK/AP-1 signaling pathway. Moreover, the restoration of various skin aging markers such as a reduction in skin wrinkle formation, skin hydration, TEWL, and HA and procollagen type I production in the dorsal skin tissues of mice also support the effect of WHS on ameliorating photo-damaged skin. These results support the argument that WHS is a potential therapeutic candidate for improving photoaged-skin.

## Supplementary Materials

The following are available online at https://www.mdpi.com/2072-6643/11/3/533/s1, Figure S1: HPLC chromatogram of WHS. Table S1: The primer sequence for qRT-PCR. Table S2: The catalog numbers and sources of antibodies for Western blot analysis.
