# Supplementary material for: Hydrangea serrata (Thunb.) Ser. Extract Attenuate UVB-Induced Photoaging through MAPK/AP-1 Inactivation in Human Skin Fibroblasts and Hairless Mice
Source: Nutrients. 2019 Mar 1;11(3):533. doi: 10.3390/nu11030533 (PMC6470489; doi:10.3390/nu11030533)
Supplement: Supplementary file 1 [file nutrients-11-00533-s001.pdf]

Table S1. The primer sequence for qRT-PCR

Gene		Sequence
**hMMP-1**	forward	5’-GCCCAGGTATTGGAGGGGAT-3'
	reverse	5’-AGGGTACATCAAAGCCCCGA-3'
**hMMP-3**	forward	5’-TTCAGCTATTTGCTTGGGAAA-3'
	reverse	5’-CAAAACATATTTCTTTGTAGAGG-3'
**hTNF-α**	forward	5’-TCTCTCTAATCAGCCCTCTG-3'
	reverse	5’-CAGATAGATGGGCTCATACC-3'
**hIL-1β**	forward	5’-AAGTACCTGAGCTCGCCAGT-3'
	reverse	5’- AGTGGTGGTCGGAGATTCGT-3'
**hIL-6**	forward	5’-CCCCAGGAGAAGATTCCAAA-3'
	reverse	5’-TTGTTTTCTGCCAGTGCCTC-3'
**hIL-8**	forward	5’-CAAACCTTTCCACCCCAAAT-3'
	reverse	5’-ACCCTCTGCACCCAGTTTTC-3'
**hGAPDH**	forward	5’-ATCAAGTGGGGCGATGCTG-3'
	reverse	5’-ACCCATGACGAACATGGGG-3'
**mIL-1β**	forward	5’-ACCTGCTGGTGTGTGACGTT-3'
	reverse	5’-TCGTTGCTTGGTTCTCCTTG-3'
**mIL-6**	forward	5'-GAGGATACCACTCCCAACAGACC-3'
	reverse	5'-AAGTGCATCATCGTTGTTCATACA-3'
**mβ-actin**	forward	5'-ATCACTATTGGCAACGAGCG-3'
	reverse	5'-TCAGCAATGCCTGGGTACAT-3'
**hCOL1A1**	forward	5’-AGGGCCAAGACGAAGACATC-3'
	reverse	5’-AGATCACGTCATCGCACAACA-3'

Table S2. The catalog numbers and sources of antibodies for Western blot analysis.

Protein	Catalog No.	Source
p-c-fos	5348S	Cell Signaling Technology
p-STAT1 (S727)	8826S	
p-p38	9215L	
Histone H3	9715	
c-fos	sc-253	Santa Cruz Biotechnology
p-c-jun	sc-822	
c-jun	sc-74543	
STAT1	sc-592	
p38	sc-271120	
p-JNK	sc-6254	
JNK	sc-7345	
p-ERK	sc-7383	
ERK	sc-94	
β-actin	sc-81178	
MMP-1	634701	Biolegend
MMP-3	634901	

Figure S1.

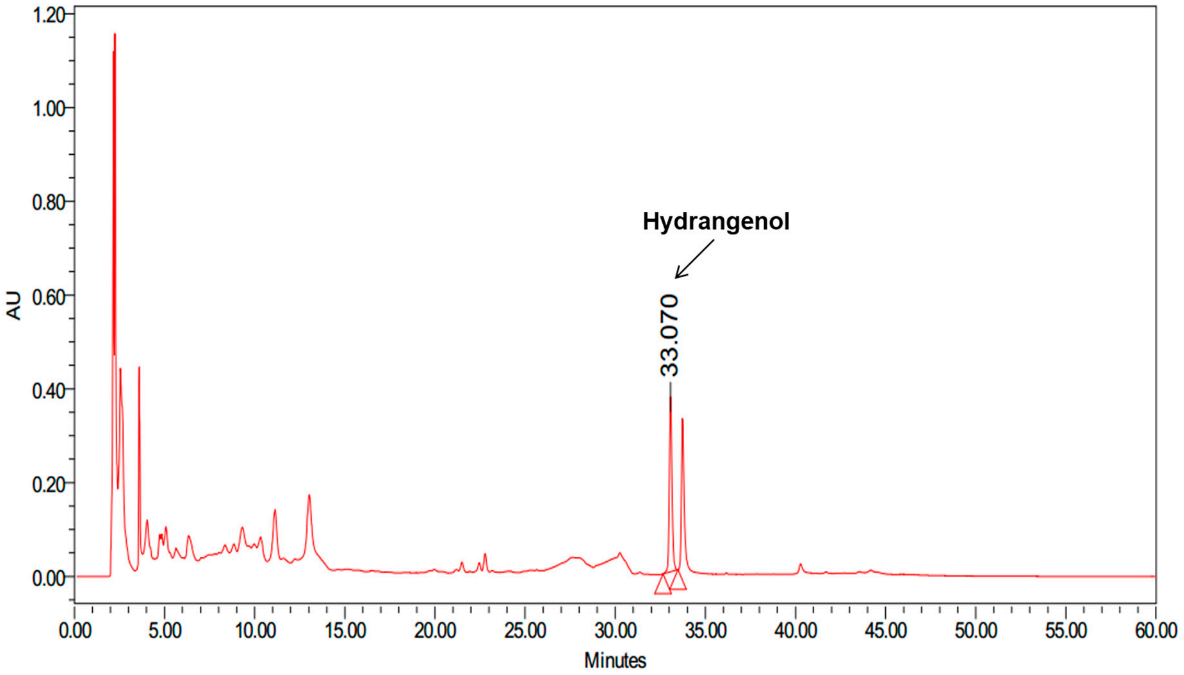

Figure S1. HPLC chromatogram of *Hydrangea serrata* (Thunb.) Ser (WHS).
